# High normal TSH is associated with lower mannan-binding lectin in women of childbearing age

**DOI:** 10.1186/s12902-019-0484-y

**Published:** 2020-01-03

**Authors:** Malgorzata Karbownik-Lewinska, Jan Stepniak, Magdalena Marcinkowska, Adrian Krygier, Andrzej Lewinski

**Affiliations:** 1 Department of Endocrinology and Metabolic Diseases, Polish Mother’s Memorial Hospital – Research Institute, 7/9 Zeligowski St., 90-752 Lodz, Poland; 2 Department of Oncological Endocrinology, Medical University of Lodz, Lodz, Poland; 3 Oxidative Stress Laboratory of the Center of Medical Laboratory Diagnostics and Screening, Polish Mother’s Memorial Hospital - Research Institute, Lodz, Poland; 4 Department of Endocrinology and Metabolic Diseases, Medical University of Lodz, Lodz, Poland

**Keywords:** Mannan-binding lectin (MBL), Thyroid tests, Euthyroid, Childbearing age, Lipid profile

## Abstract

**Background:**

Mannan-binding lectin (MBL) is a main component of the lectin pathway of the complement system. Lower MBL levels are associated with, among other conditions, hypothyroidism and adverse pregnancy outcomes. In turn, adverse pregnancy outcomes and infertility may result from hypothyroidism, even in patients with high normal Thyroid-stimulating hormone (TSH). The aim of this study was to determine if MBL level differs between women of reproductive age with low normal (< 2.5 mIU/l) and high normal (≥2.5 mIU/l) TSH. Associations with other parameters potentially affected by hypothyroidism were also evaluated.

**Methods:**

Ninety five (95) patients with normal thyroid tests (TSH 0.27–4.2 mIU/l), aged 18–48 years, were prospectively enrolled. Several laboratory parameters were measured, including MBL level, thyroid tests and lipid profile.

**Results:**

Serum MBL level was lower in women with TSH ≥ 2.5 mIU/l than with TSH < 2.5 mIU/l. This association was confirmed by univariate regression analysis. MBL level was significantly lower in patients with abnormally low HDLC/cholesterol ratio and a positive correlation was found between MBL level and HDL/cholesterol ratio.

**Conclusion:**

In women of reproductive age with normal thyroid tests, lower MBL is associated with high normal TSH and with less favourable lipid profile. Therefore treatment with L-thyroxine should be considered in women of reproductive age with TSH ≥ 2.5 mIU/l.

## Background

Mannan-binding lectin (MBL, also referred to as mannose-binding lectin) is a main component of the lectin pathway of the complement system which plays a crucial role in the immune response. According to current knowledge serum MBL level is primarily genetically determined. Other factors affecting MBL synthesis include infection, particularly sepsis [1], and hormones, including thyroid hormones [2]. In in vitro studies, thyroid hormones have been shown to increase MBL synthesis [2]. In turn, studies performed in humans revealed a clear association of MBL level with thyroid dysfunction. Namely, that an increased MBL blood level was found in hyperthyroid patients while decreased MBL levels were found in hypothyroid patients [3, 4] (hypothyroid pregnant patients included) [5]. Of great importance are findings showing that functional deficiency of MBL is associated with adverse pregnancy outcomes [6] and that MBL deficiency is found more frequently in premature neonates [7].

Taking into account the above relationship it is worth noting that hypothyroidism causes infertility and contributes to adverse pregnancy outcomes. This outcome has been repeatedly documented in human as well as in experimental studies [8]. Therefore, hypothyroid patients planning a pregnancy or who are currently pregnant should be treated with L-thyroxine.

A point of debate exists around what the thyroid-stimulating hormone (TSH) cut-off level for L-thyroxine treatment should be. Keeping TSH below 2.5 mIU/l during the pregnancy and preconception period has been recommended by different international guidelines (e.g. [9]). This was based, among other considerations, on an increased risk of miscarriage in pregnant women with TSH ≥ 2.5 mIU/l (high normal TSH) in the first trimester [10], as well as on reduced fertility rate in women with TSH ≥ 2.5 mIU/l [11], although results of some studies do not confirm that association [12]. However, the most recent guidelines by The American Thyroid Association [13], while not changing the above recommendations radically, accept high normal TSH in some patients who are pregnant or planning pregnancy.

Thus, there is still a debate in the literature concerning the upper TSH normal limit in women of childbearing age, especially at preconception and during pregnancy. We have just recently documented that in such a group of euthyroid (all thyroid tests were in normal ranges) female patients there is a relationship between TSH above 2.5 mIU/l and lipid peroxidation, as well as between TSH above 2.5 mIU/l and a less favourable lipid profile. Such findings suggest that TSH lower than 2.5 mIU/l should be kept in most women of reproductive age [14]. However, additional arguments are required to confirm that L-thyroxine treatment should be started in patients with high normal TSH. Therefore, in the present study, we have measured MBL level in the same population sample as it is another parameter affected by thyroid dysfunction [14].

The aim of the study was to determine if MBL level differs between women of reproductive age with low normal (< 2.5 mIU/l) and high normal (≥2.5 mIU/l) TSH concentration. The association of MBL with other parameters potentially affected by thyroid function was also evaluated.

## Methods

The Ethical Committee of the Polish Mother’s Memorial Hospital – Research Institute, Poland, approved all the procedures, applied in the present study, and fully informed, written consent was obtained from all patients (No. 34/2016).

Characteristics of patients enrolled into the study, as well as all indispensable procedures, were described in details elsewhere [14]. From ninety nine (99) euthyroid female inpatients with thyroid tests in reference ranges (TSH 0.27–4.2 mIU/l; free thyroxine (FT4) 0.93–1.7 ng/dl; free triiodothyronine (FT3) 2.6–4.4 pg/ml), which were considered in the previous analysis [14], ninety five (95) patients aged 18–48 years, were considered in the present statistical analysis, which did not change the basic results significantly.

The patients were divided into two groups of seventy (70) patients with low normal TSH (< 2.5 mIU/l) (Controls), and twenty five (25) patients (26.3% of the whole sample examined) with high normal TSH (≥2.5 mIU/l), which were well matched at baseline in terms of age and body mass index (BMI) (Table 1).

**Table 1:** Mean (±SEM) values of clinical/laboratory parameters in patients with TSH < 2.5 mIU/l and in patients with TSH ≥ 2.5 mIU/l. Statistical evaluation was performed by an unpaired Student’s t-test

Clinical/laboratory parameters	TSH < 2.5 mIU/l *n* = 70	TSH ≥ 2.5 mIU/l *n* = 25	*p*
Age [years]	30.95 ± 1.01	28.36 ± 1.43	0.175
Body mass [kg]	71.64 ± 2.20	74.13 ± 4.47	0.586
Height [m]	*1.66* ± 0.006	*1.64* ± 0.013	0.107
BMI [kg/m^2^]	25.85 ± 0.78	27.42 ± 1.42	0.327
Waist circumference [cm]	*82.04* ± *1.83*	*85.04* ± 3.66	0.429
Hip circumference [cm]	*103.26* ± 1.51	*104.76* ± 2.87	0.625
WHR	*0.79* ± *0.008*	*0.81* ± *0.02*	0.365
FT4 [ng/dl]	*1.23* ± *0.02*	*1.22* ± *0.03*	0.765
FT3 [pg/ml]	*2.91* ± *0.06*	*3.09 +* ±*0.07*	0.101
TPOAb [IU/ml]	*86.02* ± *18.74*	*31.27* ± *14.58*	0.097
TgAb [IU/ml]	*82.16* ± 16.81	*77.91* ± 29.56	0.898
TSHRAb [IU/l]	*0.45* ± *0.035*	*0.37* ± *0.03*	0.185
Cholesterol [mg/dl]	*176.96* ± *3.85*	*179.36* ± *6.28*	0.748
HDLC [mg/dl]	*57.06* ± *1.62*	*53.84* ± *2.90*	0.319
LDLC [mg/dl]	*96.41* ± *3.21*	*101.84* ± *5.79*	0.398
HDLC/Cholesterol	*0.33* ± *0.01*	*0.31* ± *0.02*	0.281
TGs [mg/dl]	*112.03* ± *7.98*	*113.40* ± *15.63*	0.933
Glucose [mg/dl]	*83.01* ± *0.83*	*84.20* ± *1.54*	0.479
CRP [mg/dl]	*0.76* ± *0.05*	*0.96* ± *0.16*	0.093
Iron [μg/dl]	*114.07* ± *4.82*	*102.76* ± *8.37*	0.237
Lipid peroxidation [MDA + 4-HDA (nmol/ml)]	*2.53* ± *0.09*	*2.96* ± *0.16*	0.020
MBL [ng/ml]	*1240.26* ± 63.95	*984.93* ± 85.18	0.034

Nineteen patients were previously diagnosed to be hypothyroid, therefore they were treated with L-thyroxine (25–150 μg daily).

### Parameters measured in blood serum

Apart from hormones (TSH, FT4, FT3), thyroid antibodies (i.e. thyroid peroxidase antibodies (TPOAb), thyroglobulin antibodies (TgAb) and thyrotropin receptor antibodies (TSHRAb)), among other laboratory parameters (such as total cholesterol, high-density lipoprotein cholesterol (HDLC), low-density lipoprotein cholesterol (LDLC), HDLC/cholesterol ratio, triglycerides (TGs), glucose, C-reactive protein (CRP), iron), and lipid peroxidation, were measured in blood serum and described in our previous paper [14]. Additionally, the level of Mannan Binding Lectin (MBL) was measured in the current study.

### Assay to measure MBL

Serum concentration of MBL was measured by an enzyme-linked immunosorbent assay (ELISA) using a commercial *Human MBL ELISA KIT* (RayBiotech, Inc.), with a detection threshold of 0.03 ng/ml. The protocol for the ELISA was performed following the manufacturer’s guidelines. The readings were performed on a microplate reader (Synergy H1, BioTek) with a wavelength of 450 nm.

### Statistical analysis

We have statistically analyzed the data using Student’s unpaired *t* test. We have presented the results as means ± SEM. To work out which continuous variable might have been associated with TSH ≥ 2.5 mIU/l or with L-thyroxine replacement therapy, univariate and multivariate logistic regression analyses were applied. To evaluate correlations between MBL level and all other linear parameters, Pearson’s correlation coefficient was applied. Statistical significance has been determined at the level of *p* < 0.05.

## Results

**Fig. 1:**
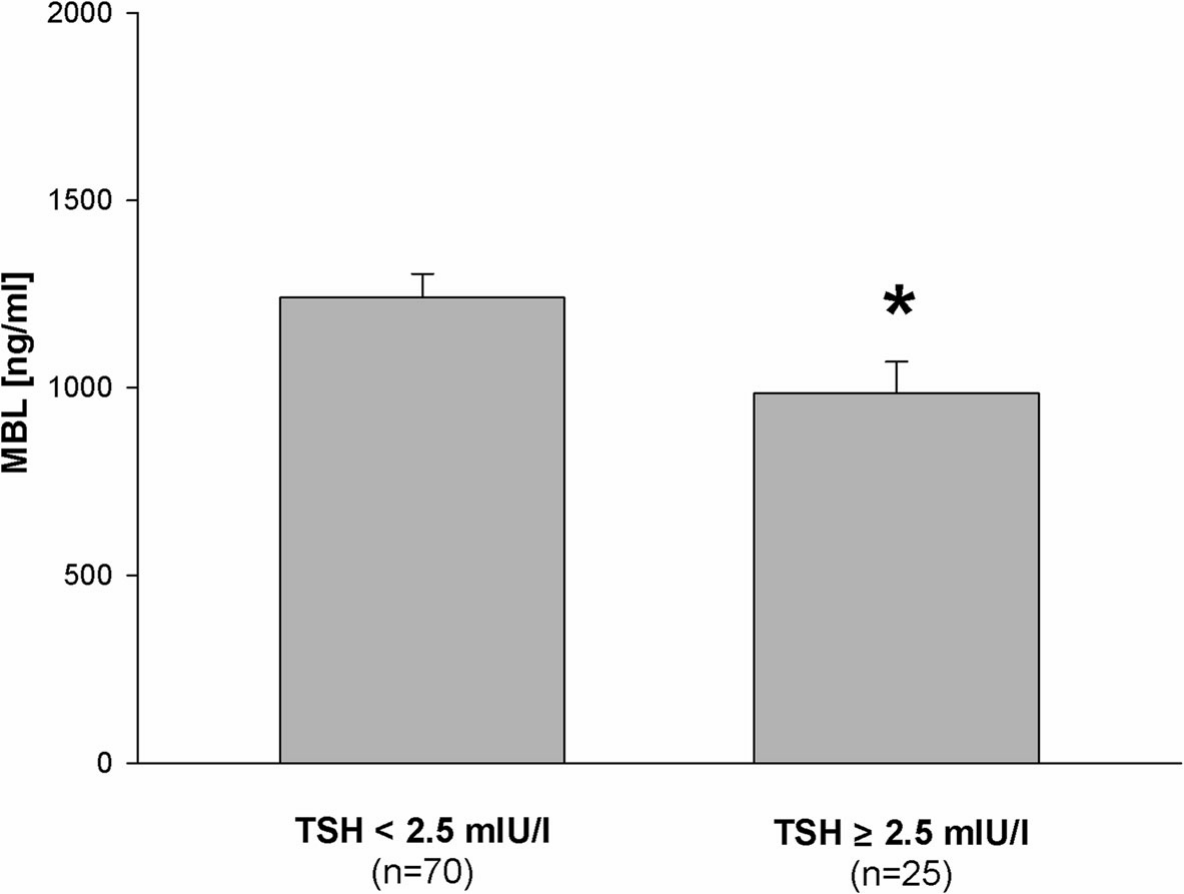
Mean (± SEM) values of MBL level in patients with TSH < 2.5 mIU/l and in patients with TSH ≥ 2.5 mIU/l. Statistical evaluation was performed by an unpaired Student’s t-test. *p < 0.05 vs. patients with TSH < 2.5 mIU/l

The main observation of the present study is that blood MBL level in euthyroid women of childbearing age was lower in a group with TSH ≥ 2.5 mIU/l than in a group with TSH < 2.5 mIU/l (Fig. 1).

**Fig. 2:**
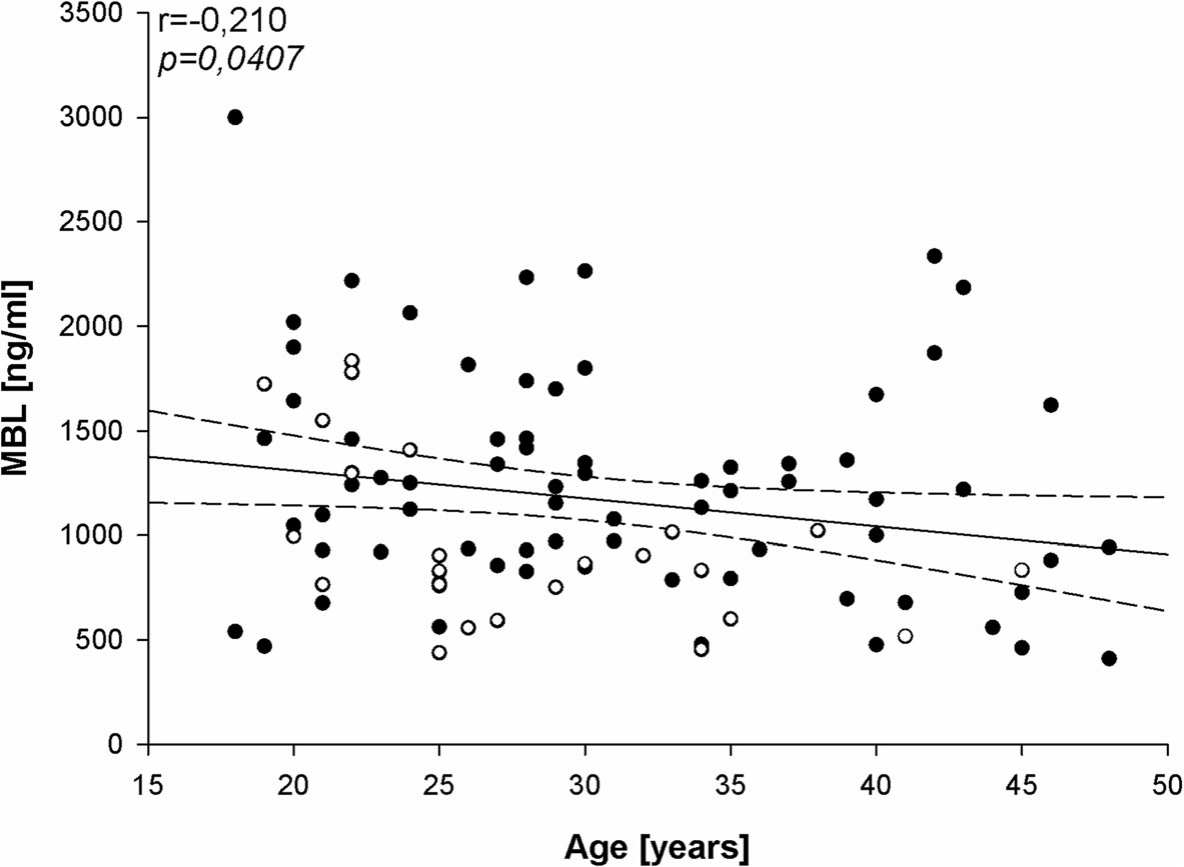
Correlation between MBL level (ng/ml) and age (years) performed in women of child-bearing age (*n* = 95). Patients with TSH < 2.5 mIU/l are marked by black dots and patients with TSH ≥ 2.5 mIU/l are marked by circles; *r* = Pearson’s correlation coefficient; *p < 0.05

**Fig. 3:**
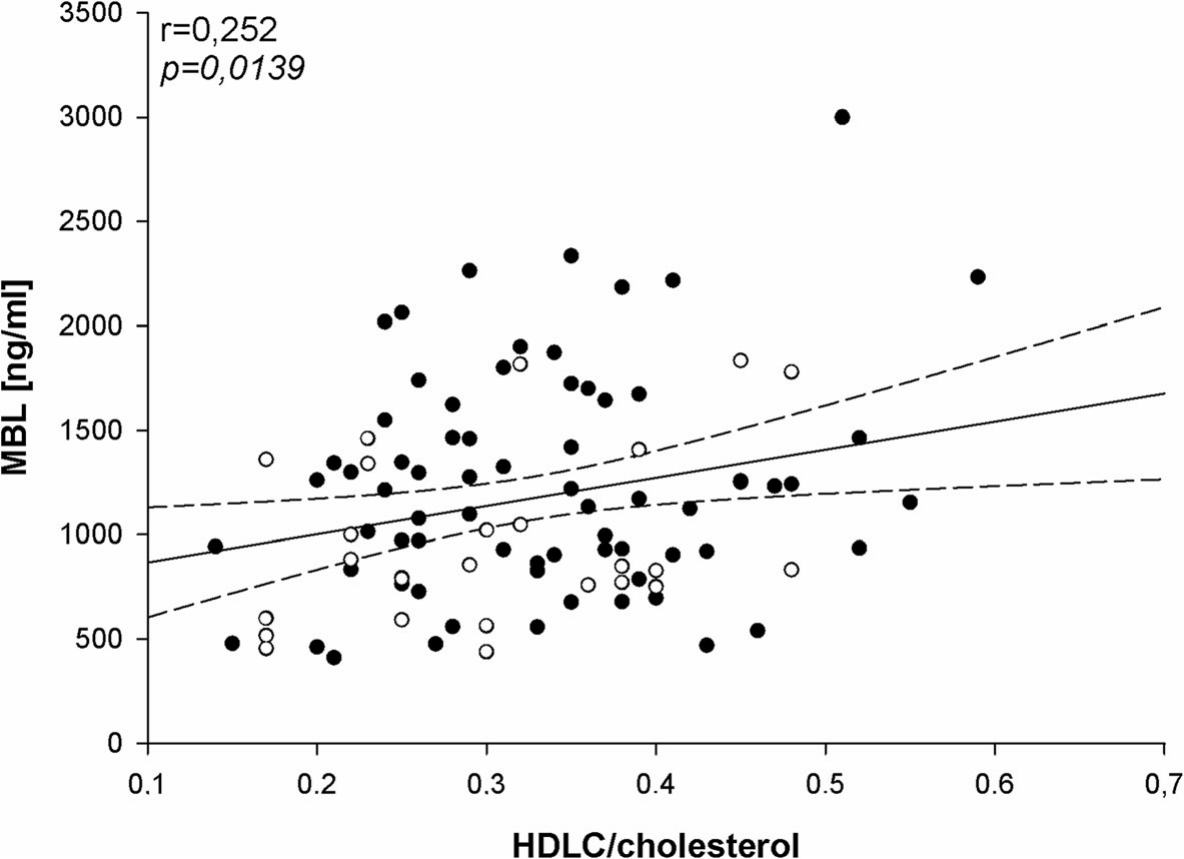
Correlation between MBL level (ng/ml) and HDLC/cholesterol performed in women of child-bearing age (*n* = 95). Patients with TSH < 2.5 mIU/l are marked by black dots and patients with TSH ≥ 2.5 mIU/l are marked by circles; *r* = Pearson’s correlation coefficient; *p < 0.05

**Fig. 4:**
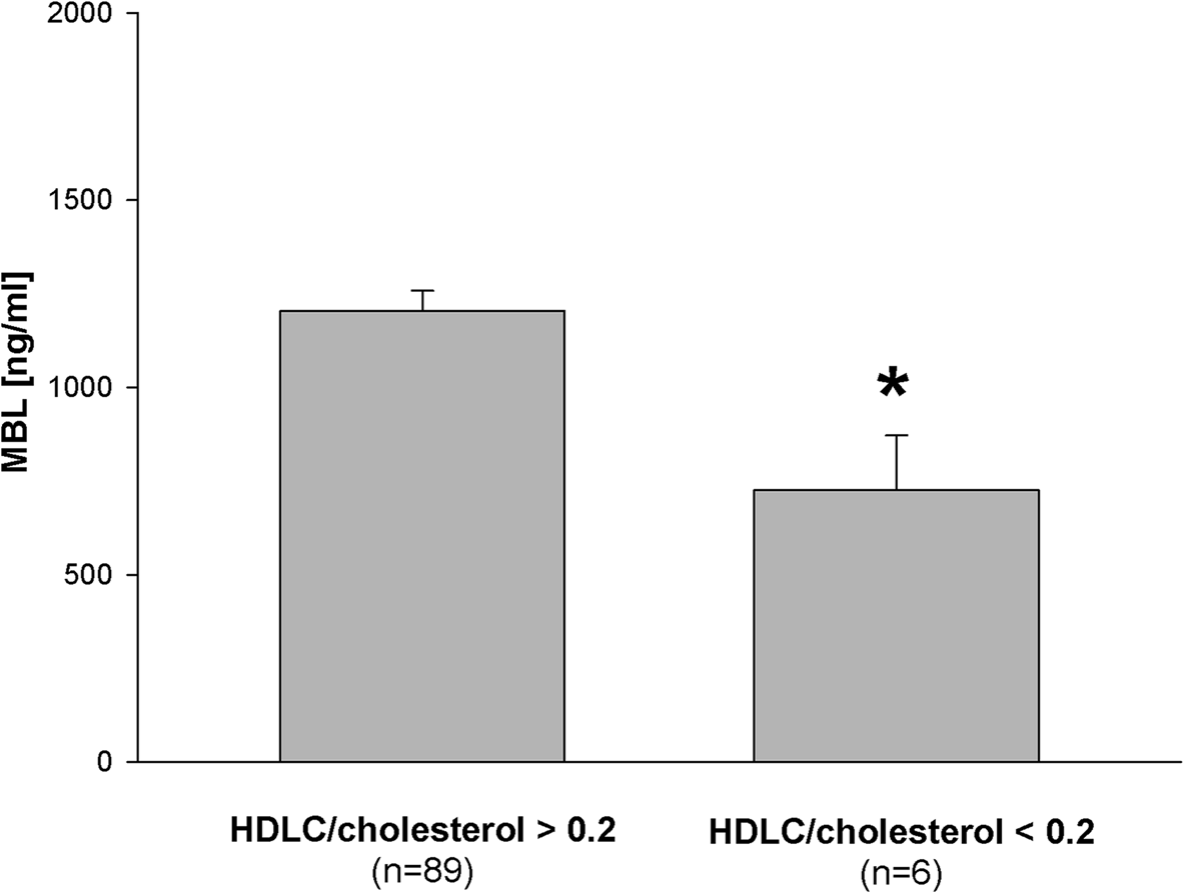
Mean (± SEM) values of MBL level in patients with HDLC/cholesterol > 0.2 and in patients with HDLC/cholesterol < 0.2. Statistical evaluation was performed by an unpaired Student’s t-test. *p < 0.05 vs. patients with HDLC/cholesterol > 0.2

We have found negative correlation between MBL level and age (Fig. 2) as well as positive correlation between MBL level and HDL/cholesterol ratio (Fig. 3). In agreement with the last finding, MBL level was significantly lower in patients with abnormally low HDLC/cholesterol ratio (Fig. 4).

MBL level did not correlate with either TPOAb (*r* = 0.0520, *p* = 0.621) or TgAb (*r* = 0.1216, *p* = 0.246). Accordingly, the group with positive thyroid antibodies (31 patients with positive either TPOAb or TgAb or both) and the group with negative thyroid antibodies (mean ± SEM; 1170.34 ± 88.95 ng/ml vs. 1174.38 ± 66.72 ng/ml, respectively; *p* = 0.972) were characterized by similar MBL levels (no statistically significant differences were found between groups). Similarly, when TPOAb and TgAb were considered separately, MBL level did not differ statistically between groups with abnormally high and normal thyroid antibodies (data not shown).

In our previous study [14] we found in the univariate regression analysis that among all measured linear parameters (variables) only blood lipid peroxidation level was statistically associated with TSH ≥ 2.5 mIU/l. In the present regression analysis we added the additional variable, blood MBL level, and have found that both blood lipid peroxidation level and blood MBL level were statistically associated with high normal TSH (Table 2). However, in multivariate regression analysis, MBL level lost its statistical significance and lipid peroxidation level was confirmed to be the only independent factor associated with TSH ≥ 2.5 mIU/l (Table 3). The results of the regression analysis confirmed that patients with TSH ≥ 2.5 mIU/l and TSH < 2.5 mIU/l were matched in terms of age and BMI.

**Table 2:** Univariate logistic regression analysis of the univariate increased TSH (for TSH ≥ 2.5 mIU/l) determinants (variables), performed in women of child-bearing age (n = 95). OR, odds ratio; CI, confidence interval; the level of statistical significance is given in italic; *p < 0.05

Variable	Univariate regression		
	OR	95%Cl	*p*
Age [years]	0.959	0.90–1.02	*0.175*
Body mass [kg]	1.006	0.98–1.03	*0.581*
Height [m]	0.0012	2.9 × 10^−7^-5.15	*0.110*
BMI [kg/m^2^]	1.033	0.97–1.10	*0.326*
Waist circumference [cm]	1.011	0.98–1.04	*0.425*
Hip circumference [cm]	1.009	0.97–1.04	*0.615*
WHR	18.53	0.032–10,625.13	*0.361*
FT4 [ng/dl]	0.684	0.05–8.24	*0.762*
FT3 [pg/ml]	2.768	0.81–9.36	*0.096*
aTPO-Ab [IU/ml]	0.995	0.98–1.00	*0.131*
aTg-Ab [IU/ml]	0.999	0.99–1.00	*0.897*
TSHR-Ab [IU/l]	0.199	0.02–2.48	*0.203*
Cholesterol [mg/dl]	1.002	0.98–1.02	*0.745*
HDLC [mg/dl]	0.982	0.94–1.01	*0.315*
LDLC [mg/dl]	1.007	0.99–1.02	*0.395*
HDLC/Cholesterol	0.065	0.0004–9.71	*0.278*
TGs [mg/dl]	1.000	0.99–1.00	*0.932*
Glucose [mg/dl]	1.014	0.95–1.08	*0.663*
CRP [mg/dl]	1.951	0.83–4.53	*0.115*
Iron [μg/dl]	0.993	0,98–1.01	*0.235*
Lipid peroxidation [MDA + 4-HDA (nmol/ml)]	1.997	1.08–3.66	0.023*
MBL [ng/ml]	0.999	0.998–0.999	0.038*

**Table 3:** Multivariate logistic regression analysis of the univariate increased TSH (for TSH ≥2.5 mIU/l) determinants (variables), performed in women of child-bearing age (*n* = 95). OR, odds ratio; CI, confidence interval; the level of statistical significance is given in italic; *p < 0.05

Variable	Multivariate regression		
	OR	95%Cl	*p*
Lipid peroxidation [MDA + 4-HDA (nmol/ml)]	1.945	1.04–3.62	*0.033*
MBL [ng/ml]	0.998	0.99–1.00	*0.052*

MBL level was not statistically associated with L-thyroxine treatment when evaluated by the univariate logistic regression analysis (OR = 0.999; CI = 0.998–1.000, *p =* 0.797). Consistently, MBL level in patients on L-thyroxine replacement (*n* = 19) did not differ from MBL level in patients not treated with L-thyroxine (*n* = 76) (mean ± SEM; 1145.89 ± 122.90 ng/ml vs. 1179.86 ± 59.42 ng/ml, respectively; *p* = 0.800.

## Discussion

In our present analysis we have found another argument, not associated directly with pregnancy or fertility, for keeping TSH below 2.5 mIU/l, or at least in lower normal ranges, in women of childbearing age.

We have observed that MBL level, being a component of innate immune response, is lower in female patients of reproductive age having high normal TSH. Additionally, lower levels of MBL were associated with a worse lipid profile, which was confirmed by a lower MBL level in patients with abnormally low HDLC/cholesterol ratio and by positive correlation between MBL level and HDLC/cholesterol ratio.

Genetically determined reference ranges of MBL are extremely broad. However, in the case of identical genotypes, MBL synthesis may change in response to different pathological conditions, as was mentioned in the Introduction section. Concerning relationship between MBL level and thyroid function, lower MBL values were linked to hypothyroidism [3–5]. Thus, our present observation showing lower MBL concentrations in women with TSH ≥ 2.5 mIU/l suggests that high normal TSH in women at childbearing age is less favorable and, therefore, should be considered abnormal.

The other argument arising from the present study for keeping TSH in lower normal reference ranges relates to the identification of less favorable lipid profiles in patients with lower MBL level. This is in agreement with our previous results [14] showing that women with TSH ≥ 2.5 mIU/l have less favorable lipid profiles which is in turn associated with higher oxidative damage to membrane lipids. This is also in line with suggestion to substantially decrease the TSH cut-off for diagnosing of Hashimoto thyroiditis [15].

As we documented that higher lipid peroxidation level [14] and lower MBL level (present study) were found to be associated with TSH ≥ 2.5 mIU/l, we decided to statistically evaluate which of them is a greater determinant of high normal TSH. When evaluated by univariate logistic regression analysis, among numerous linear variables, lipid peroxidation level and MBL level were the only two determinants associated with higher normal TSH concentration. However, multivariate logistic regression analysis revealed that lipid peroxidation level is the only independent factor associated with TSH ≥ 2.5 mIU/l. Thus, on the basis of our results it may be proposed that oxidative damage is more strongly associated with abnormal TSH when compared to immune response, specifically the lectin pathway of the complement system.

However, our results are still of great value because they show abnormal immune responses resulting from higher, but normal, TSH. Thus, our results open a new field of immune mechanisms responsible for different organ abnormalities in response to mild thyroid dysfunction. It can be hypothesized that certain abnormalities of the lectin pathway of the complement system are already present in conditions of mild hypothyroidism, and that they can contribute to such events as infertility or adverse pregnancy outcome.

We have confirmed in the present study that MBL level did not depend on the presence of thyroid antibodies. This finding is of great value as both, i.e. MBL and thyroid antibodies, are components of the immune system. Thus, our results confirm that MBL level is affected by thyroid dysfunction independently of autoimmune process, the latter being the principal etiological factor of hypothyroidism.

Similarly, we have observed in the present study that MBL level was not statistically associated with L-thyroxine treatment, which again underlines strong associations between MBL level and thyroid function.

We have found in our study that MBL level correlates negatively with age. This observation was expected, as immune system efficiency decreases naturally with age, and is in agreement with earlier observations showing that the lectin pathway activated by mannan is lower in the elderly [16].

## Conclusions

In women of reproductive age showing normal thyroid tests, TSH in the upper normal range was associated with lower MBL level. This relationship suggests that abnormal immune response may result from high normal TSH, and thereafter it may potentially affect different physiological processes, such as fertility. Additionally, lower MBL concentration was associated with a less favorable lipid profile, which suggests the potential contribution of the former to proatherogenic effects, in turn resulting in a decline of general health. Therefore, our results support the point of view that TSH higher than 2.5 mIU/l is associated with certain abnormalities in women of reproductive age and, therefore, treatment with L-thyroxine should be considered in these patients. Our study is the first one to document that TSH ≥ 2.5 mIU/l in women of childbearing age is associated with some abnormalities in the lectin pathway of the complement system.

## Data Availability

The datasets used and/or analyzed during the current study are available from the corresponding author on reasonable request.

## Abbreviations

CRP: C-reactive protein
ELISA: Enzyme-linked immunosorbent assay
FT3: Free triiodothyronine
FT4: Free thyroxine
HDL: High-density lipoprotein
LDL: Low-density lipoprotein
MBL: Mannan-binding lectin
TG: Triglycerides
TgAb: Thyroglobulin antibodies
TPOAb: Thyroid peroxidase antibodies
TSH: Thyroid-stimulating hormone
TSHRAb: Thyrotropin receptor antibodies
