# Correction to: High normal TSH is associated with lower mannan-binding lectin in women of childbearing age

**DOI:** 10.1186/s12902-020-00564-x

**Published:** 2020-06-09

**Authors:** Malgorzata Karbownik-Lewinska, Jan Stepniak, Magdalena Marcinkowska, Adrian Krygier, Andrzej Lewinski

**Affiliations:** 1 Department of Endocrinology and Metabolic Diseases, Polish Mother’s Memorial Hospital – Research Institute, Lodz, Poland; 2 Department of Oncological Endocrinology, Medical University of Lodz, Lodz, Poland; 3 Oxidative Stress Laboratory of the Center of Medical Laboratory Diagnostics and Screening, Polish Mother’s Memorial Hospital -Research Institute, Lodz, Poland; 4 Department of Endocrinology and Metabolic Diseases, Medical University of Lodz, Lodz, Poland

**Correction to: BMC Endocrine Disorders (2020) 20:1**

**https://doi.org/10.1186/s12902-019-0484-y**

In the original publication of this article [1] the first affiliation of the authors is incorrect, the correct affiliation of the first one is: Department of Endocrinology and Metabolic Diseases, Polish Mother’s Memorial Hospital – Research Institute, Lodz, Poland.
